# Supplementary material for: A noncoding variant confers pancreatic differentiation defect and contributes to diabetes susceptibility by recruiting RXRA
Source: Nat Commun. 2024 Nov 12;15:9771. doi: 10.1038/s41467-024-54151-y (PMC11557932; doi:10.1038/s41467-024-54151-y)
Supplement: Supplementary file 1 — Supplementary Information [file 41467_2024_54151_MOESM1_ESM.pdf]

Supplementary Materials for

A noncoding variant confers pancreatic differentiation defect and contributes to diabetes susceptibility by recruiting RXRA

Yinglei Li[1], Ran Zheng[1], Lai Jiang[1], Chenchao Yan[1], Ran Liu[1], Luyi Chen[1], Wenwen Jin[1], Yuanyuan Luo[1], Xiafei Zhang[1], Jun Tang[2], Zhe Dai[2] and Wei Jiang[1,3,4,*]

1 Department of Biological Repositories, Frontier Science Center for Immunology and Metabolism, Medical Research Institute, Zhongnan Hospital of Wuhan University, Wuhan University; Wuhan, 430071, China.

2 Department of Endocrinology, Zhongnan Hospital of Wuhan University; Wuhan, China

3 Human Genetics Resource Preservation Center of Wuhan University; China.

4 Hubei Provincial Key Laboratory of Developmentally Originated Disease; China.

*Corresponding author. Email: jiangw.mri@whu.edu.cn

Supplementary Figures

Supplementary Figure 1: Editing to obtain PSCs with rs6048205 variants.

Supplementary Figure 2: PSCs with rs6048205-G formed DE and PDX1+ PP1 cells normally.

Supplementary Figure 3: rs6048205-G affected pancreatic progenitor differentiation.

Supplementary Figure 4: rs6048205-G had minimal effect on cell apoptosis.

Supplementary Figure 5: FOXA2 overexpression in PP stage affected pancreatic development *in vitro*.

Supplementary Figure 6: rs6048205-G enhanced RXRA binding.

Supplementary Figure 7: The function of rs6048205-G in the mouse model.

Supplementary Figure 8: Representative flow cytometry plots and gating strategy.

Supplementary Tables

Supplementary Table 1. Primers for RT-qPCR analyses.

Supplementary Table 2. Primers for ChIP-qPCR analyses.

Supplementary Table 3. Sequence of EMSA probes.

Supplementary Figure 1

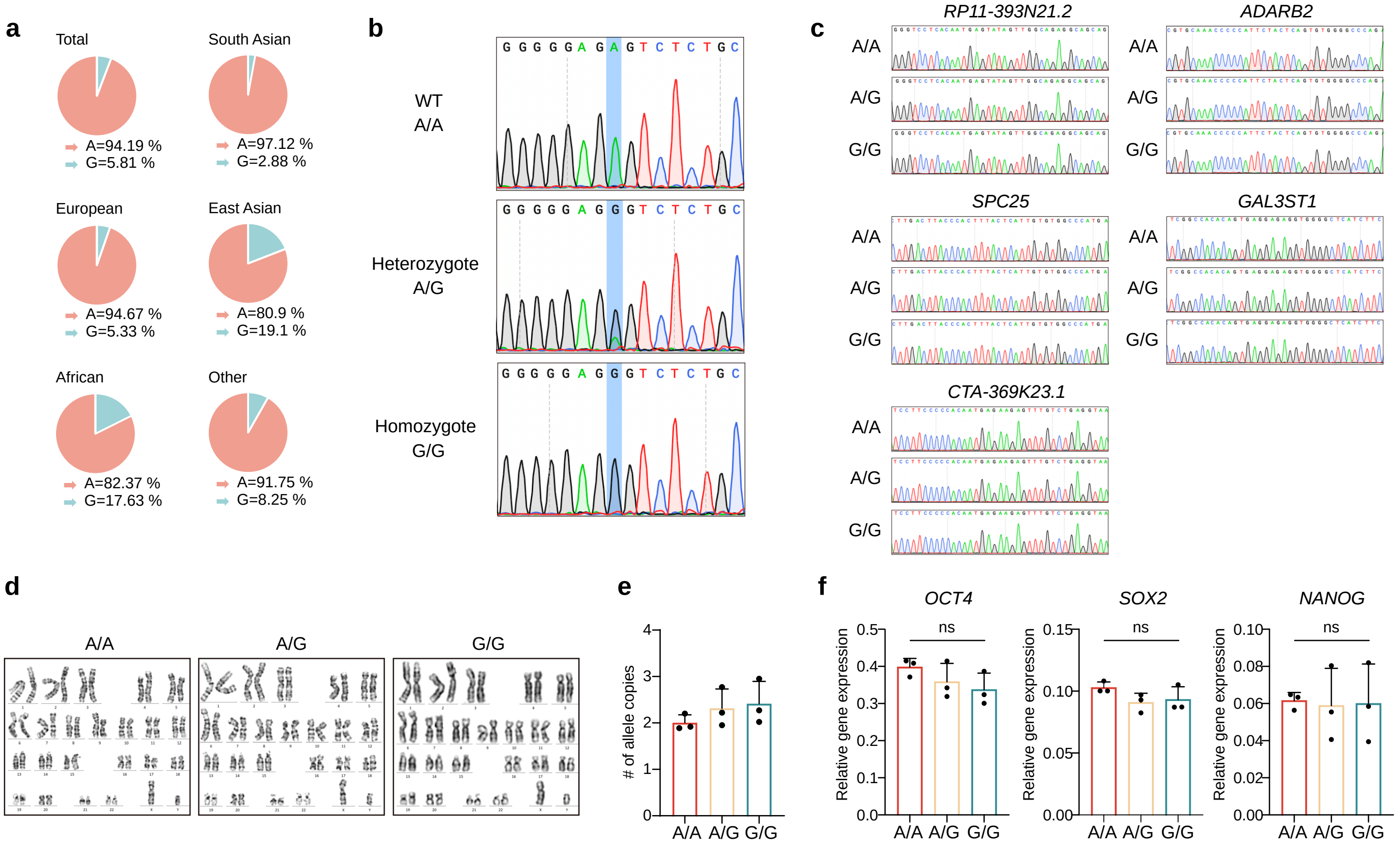

Supplementary Figure 1: Editing to obtain PSCs with rs6048205 variants.

a Allelic frequency of rs6048205 in different populations from ALFA (Allele Frequency Aggregator). **b** Sanger sequencing reads of SNP rs6048205 in CRISPR/Cas9-edited PSCs. **c** PCR amplification and Sanger sequencing of about 500 bp surrounding potential off-target regions revealed no sequence deviation in these regions in the edited PSCs. Potential off-target regions were obtained by the CCTop CRISPR/Cas9 off-target online predictor. **d** G-band karyotype analysis of edited PSCs. **e** qPCR assay assessed allele copy numbers in edited PSCs (n = 3 independent experiments). **f** mRNA expression levels of pluripotency markers (*OCT4*, *SOX2* and *NANOG*) of edited iPSCs quantified by RT-qPCR (n = 3 independent experiments). Data are presented as the mean ± SD. Statistical significance was determined using the unpaired, two-tailed t-test in **f** (ns not significant).

Supplementary Figure 2

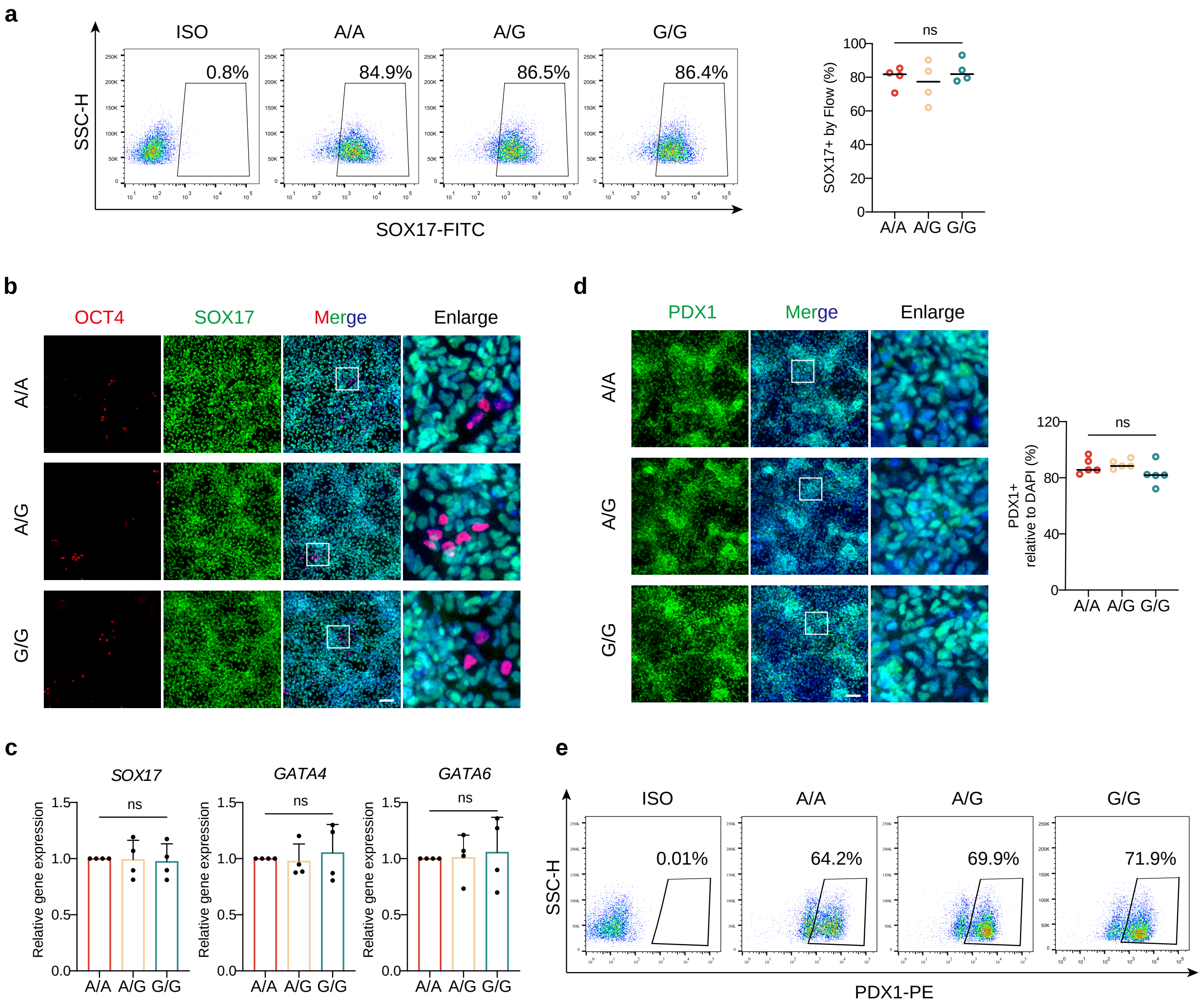

Supplementary Figure 2: PSCs with rs6048205-G formed DE and PDX1+ PP1 cells normally.

a Flow cytometry analysis of SOX17 in DE stage and statistical results (n = 4 independent experiments). **b** Immunofluorescence staining of DE cells with DE marker (SOX17) and pluripotent marker (OCT4). Scale bar, 100 μm. **c** mRNA expression levels of DE markers (*SOX17*, *GATA4* and *GATA6*) quantified by RT-qPCR (n = 4 independent experiments). Gene expression levels were normalized to *GAPDH* and then further normalized to the A/A group. **d** Immunofluorescence staining of PP1 cells with PDX1 and statistical analysis (n = 5 images). Scale bar, 100 μm. **e** Flow cytometry analysis of PDX1 in PP1 stage (n = 3 independent experiments). Data are presented as the mean ± SD. Statistical significance was determined using the unpaired, two-tailed t-test in **a**, **c**, **d** (ns not significant).

Supplementary Figure 3

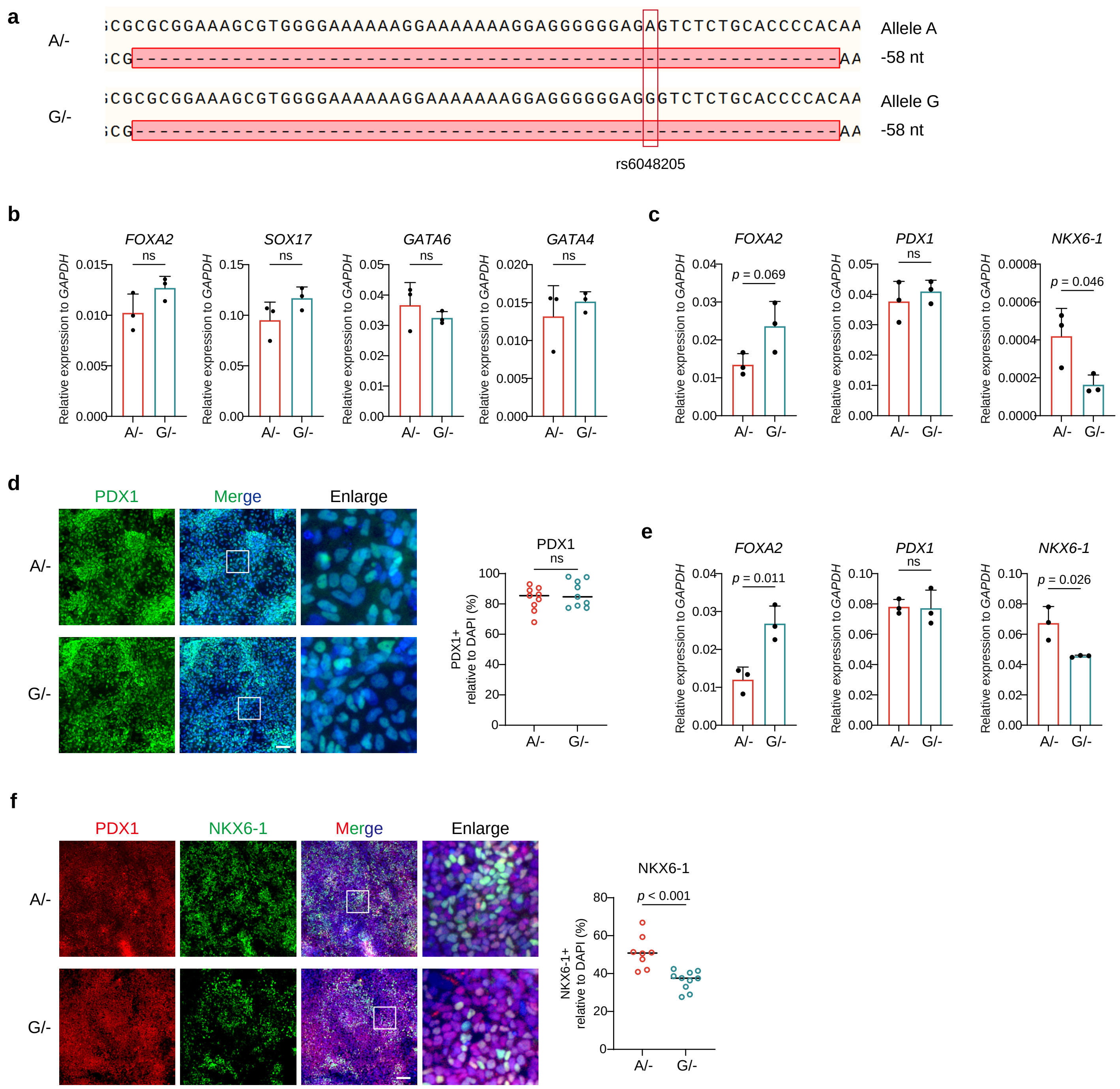

Supplementary Figure 3: rs6048205-G affected pancreatic progenitor cell differentiation.

a Genotyping of A/- and G/- cells obtained from HUES8. Each cell has a 58 nt deletion containing SNP rs6048205 on one allele. **b** mRNA expression levels of DE markers (*SOX17*, *GATA4* and *GATA6*) quantified by RT-qPCR ($n = 3$ independent experiments). **c** mRNA expression levels of *FOXA2* and PP1 markers (*PDX1* and *NKX6-1*) quantified by RT-qPCR ($n = 3$ independent experiments). **d** Immunofluorescence staining of PP1 cells with PDX1 and statistical analysis ($n = 9$ images). Scale bar, 100 µm. **e** mRNA expression levels of *FOXA2* and PP2 markers (*PDX1* and *NKX6-1*) quantified by RT-qPCR ($n = 3$ independent experiments). **f** Representative immunofluorescence images of PP2 cells stained by PDX1 (red) and NKX6-1 (green) and quantitative analysis for the percentage of NKX6-1-positive cells ($n = 9$ images). Nuclei were counterstained with DAPI (blue). Scale bar, 100 µm. Data are presented as the mean ± SD. Statistical significance was determined using the unpaired, two-tailed *t*-test in **b**-**f** (ns not significant).

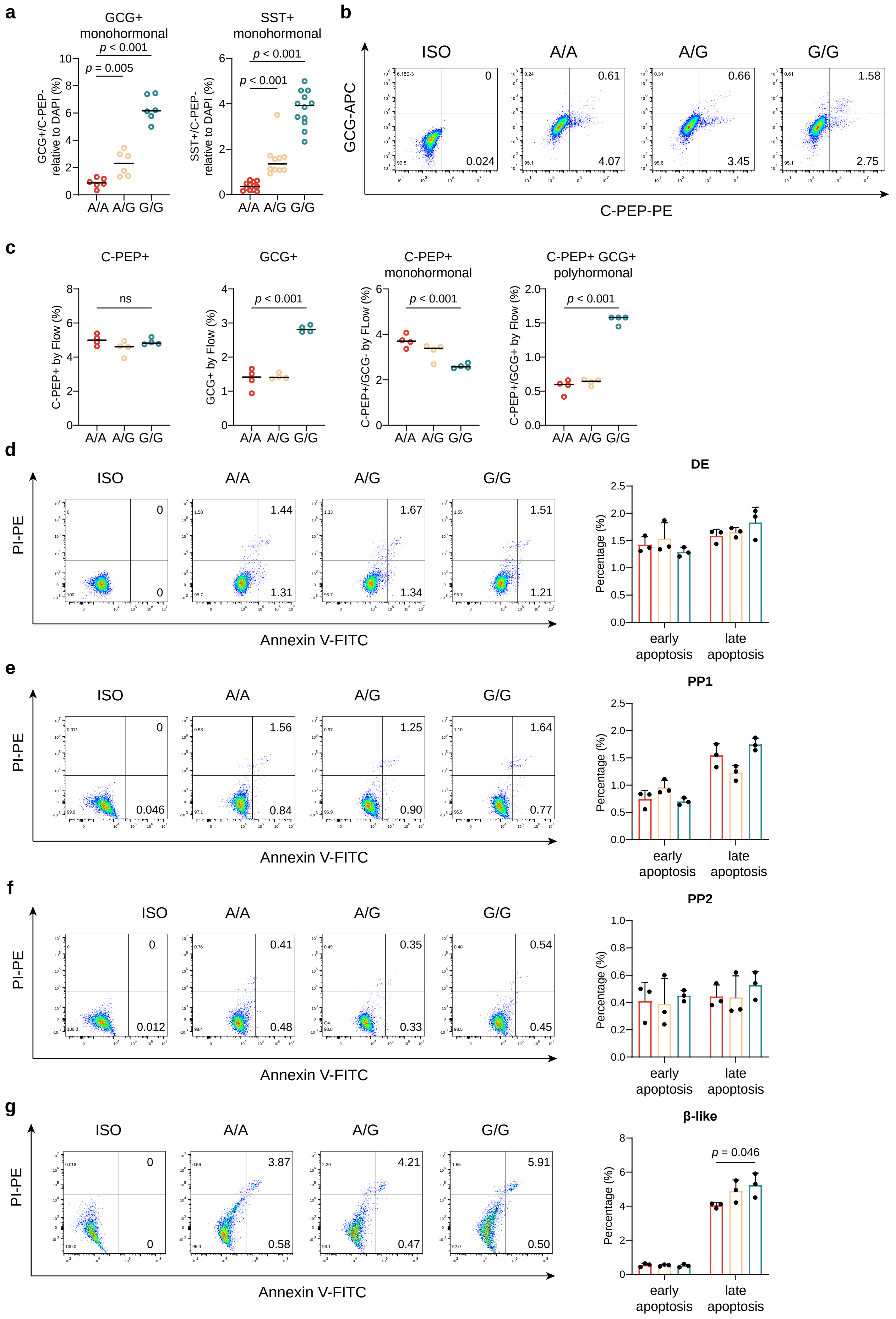
a
GCG+ monohormonal
SST+ monohormonal
p < 0.001
p = 0.005
p < 0.001
GCG+/C-PEP- relative to DAPI (%)
SST+/C-PEP- relative to DAPI (%)
A/A A/G G/G
b
ISO
A/A
A/G
G/G
GCG-APC
C-PEP-PE
0
0.024
0.61
4.07
0.66
3.45
1.58
2.75
c
C-PEP+
GCG+
C-PEP+ monohormonal
C-PEP+ GCG+ polyhormonal
ns
C-PEP+ by Flow (%)
GCG+ by Flow (%)
C-PEP+/GCG- by FLow (%)
C-PEP+/GCG+ by Flow (%)
d
PI-PE
Annexin V-FITC
0
0
1.44
1.31
1.67
1.34
1.51
1.21
DE
Percentage (%)
early apoptosis
late apoptosis
e
0
0.046
1.56
0.84
1.25
0.90
1.64
0.77
PP1
f
0
0.012
0.41
0.48
0.35
0.33
0.54
0.45
PP2
g
0
0
3.87
0.58
4.21
0.47
5.91
0.50
β-like
p = 0.046

Supplementary Figure 4: rs6048205-G had minimal effect on cell apoptosis.

a Quantitative analysis for the percentage of GCG (*n* = 6 images) and SST (*n* = 11 images in A/A, *n* = 10 in A/G and *n* = 12 in G/G) monohormonal cells in Fig.**2d-e**. **b-c** Flow cytometry analysis of C-PEP and GCG in β-like cell stage (**b**) and statistical results (**c**) for the percentage of C-PEP+, GCG+, C-PEP+ monohormonal cells and C-PEP+/GCG+ polyhormonal cells (*n* = 4 independent experiments). **d-g** Apoptosis assay with PI and Annexin V double staining detected by FACS in DE (**d**), PP1(**e**), PP2(**f**), and β-like cell (**g**) stage, the statistical results are shown right. Early apoptosis was defined as Annexin V positive, and late apoptosis was defined as PI- and Annexin V-double positive (*n* = 3 independent experiments). Data are presented as the mean ± SD. Statistical significance was determined using the unpaired, two-tailed *t*-test in **a**, **c-g** (ns not significant).

Supplementary Figure 5

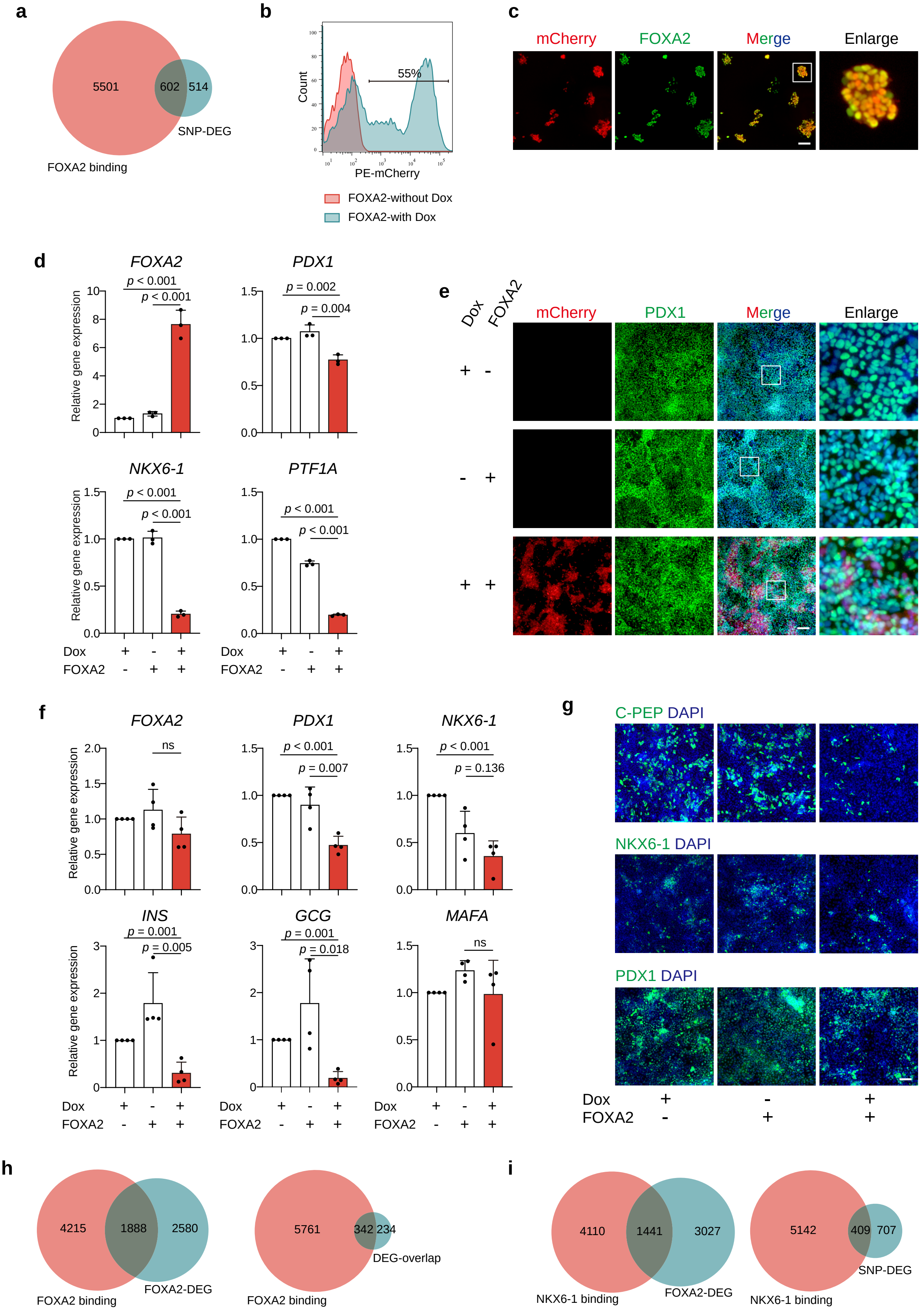

Supplementary Figure 5: FOXA2 overexpression in PP stage affected pancreatic development *in vitro*.

a Venn diagram showing differentially expressed genes (DEGs) overlap in SNP-G mutation and FOXA2 binding targets. **b** mCherry expression of inducible FOXA2 overexpressed ESCs with or without Dox quantified by flow cytometry. **c** Immunofluorescence staining of inducible FOXA2 overexpressed ESCs with FOXA2 and mCherry after 2 days of Dox treatment. Scale bar, 100 µm. **d** mRNA levels of *FOXA2* and PP2 markers (*PDX1*, *NKX6-1* and *PTF1A*) detected by RT-qPCR in PP2 stage (n = 3 independent experiments). Gene expression levels were normalized to *GAPDH* and then further normalized to the control group. **e** Immunofluorescence of PP2 cells stained by PDX1 (green), nuclei were counterstained with DAPI. Scale bar, 100 µm. **f** mRNA expression levels of β cell markers quantified by RT-qPCR (n = 4 independent experiments). Gene expression levels were normalized to *GAPDH* and then further normalized to the control group. **g** Immunofluorescence of β cells stained by PDX1, NKX6-1, and C-PEP; nuclei were counterstained with DAPI. Scale bar, 100 µm. **h** Left: Venn diagram showing the overlap of differentially expressed genes (DEGs) in FOXA2 overexpress and FOXA2 targets; right: Venn diagram showing the overlap of differentially expressed genes (DEGs) in SNP-G and FOXA2 targets. **i** Left: Venn diagram showing the overlap of differentially expressed genes (DEGs) in FOXA2 overexpress and NKX6-1 binding genes; right: Venn diagram showing the overlap of differentially expressed genes (DEGs) in SNP risk allele G group and NKX6-1 binding genes. Data are presented as the mean ± SD. Statistical significance was determined using the unpaired, two-tailed *t*-test in **d**, **e** (ns not significant).

Supplementary Figure 6

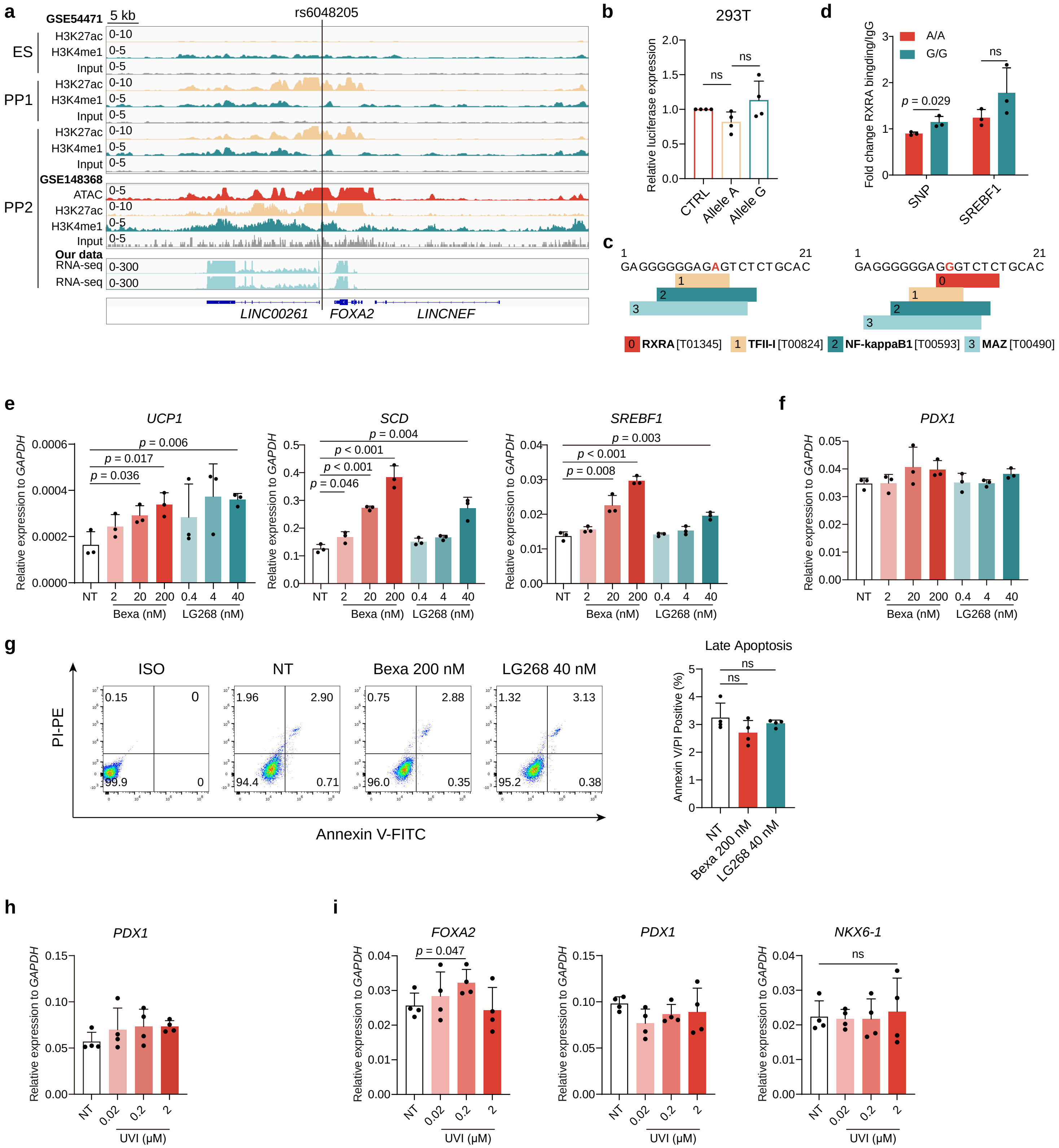

Supplementary Figure 6: rs6048205-G enhanced RXRA binding.

a Bedgraph for H3K27ac and H3K4me1 ChIP-seq, ATAC-seq and RNA-seq data at SNP rs6048205 loci. The x-axis corresponds to genomic locations with the scale at the panel's top. The y-axis corresponds to signal intensity. **b** Firefly luciferase/Renilla luciferase activity for different rs6048205 alleles in 293T cells (n = 4 independent experiments). **c** Motif prediction of sequence around rs6048205 performed using PROMO. **d** ChIP-qPCR for RXRA normalized to IgG control at rs6048205 using PP cells differentiated from the A/A and G/G cell lines (n = 3 independent experiments). **e** mRNA levels of RXRA downstream genes *UCP1*, *SCD,* and *SREBF1* in PP2 cells treated with different dosages of RXR agonists bexarotene (Bexa) and LG000268 (LG268) detected by RT-qPCR analysis (n = 3 independent experiments). **f** mRNA levels of *PDX1* in PP2 cells treated with different dosages of RXR agonists by RT-qPCR analysis (n = 3 independent experiments). **g** Apoptosis assay with PI and Annexin V double staining detected by FACS in PP2 cells treated with the highest dosage of RXR agonists. The statistical results are right: early apoptosis was defined as Annexin V positive, and late apoptosis was defined as PI and Annexin V double positive (n = 3 independent experiments). **h** mRNA levels of *PDX1* in G/G PP2 cells treated with different dosages of RXR inhibitor UVI3003 (UVI) by RT-qPCR analysis (n = 4 independent experiments). **i** mRNA levels of *FOXA2*, *PDX1* and *NKX6-1* in A/A PP2 cells treated with different dosages of RXR inhibitor UVI3003 (UVI) by RT-qPCR analysis (n = 4 independent experiments). Data are presented as the mean ± SD. Statistical significance was determined using the unpaired, two-tailed t-test in **b**, **d**, **e-i** (ns not significant).

Supplementary Figure 7

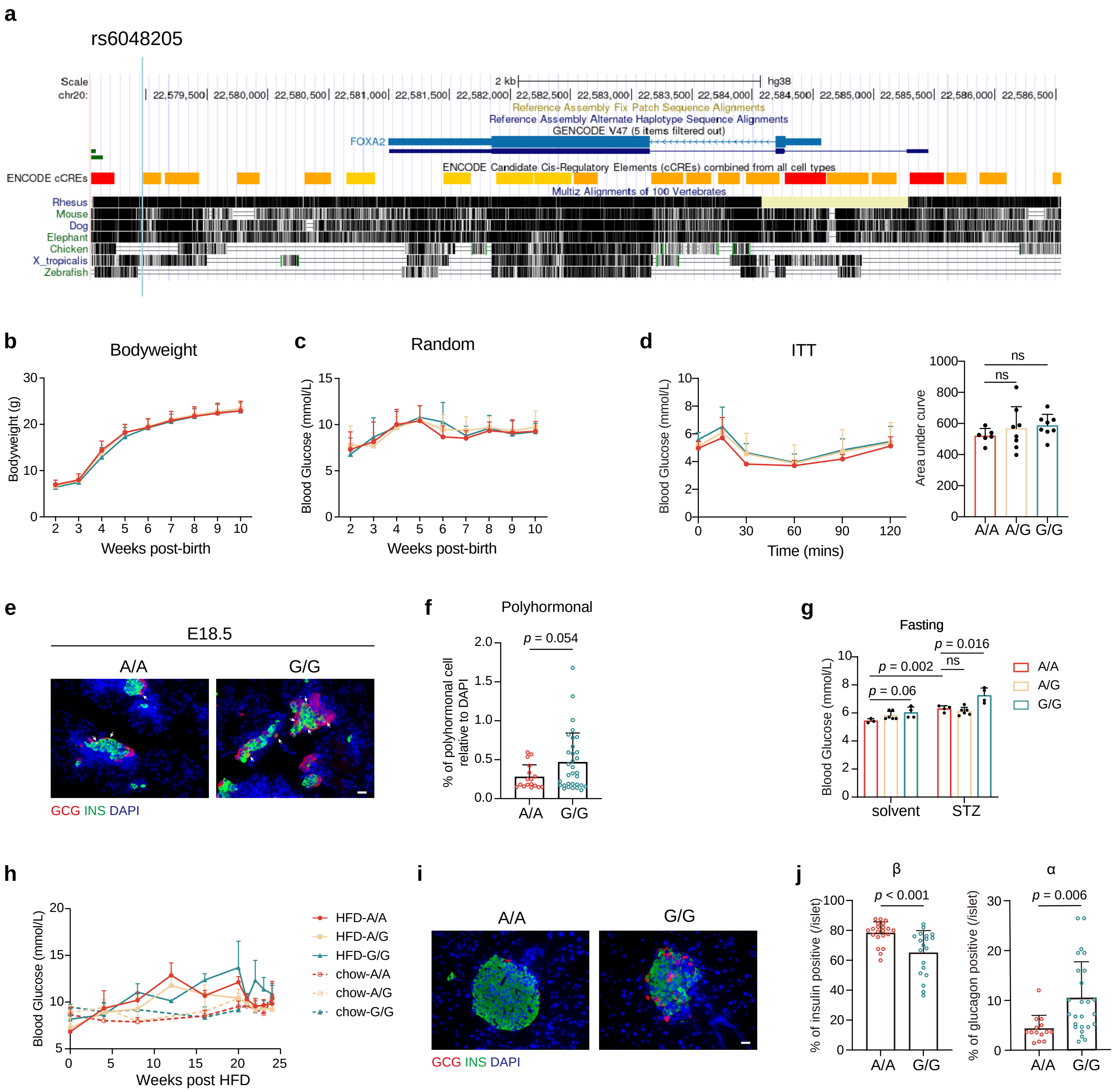

Supplementary Figure 7: The function of rs6048205 in the mouse model.

a Conservation analysis downloaded from UCSC. The SNP rs6048205 was marked by a blue line. **b** Body weight of each genotype over time post birth (n = 18 mice in A/A, n = 31 A/G and n = 16 in G/G). **c** Quantification of the blood glucose of each genotype over time (n = 18 mice in A/A, n = 31 in A/G and n = 16 in G/G). **d** Insulin tolerance tests (ITT) were performed in fasting 6 hours. The area under the curve (AUC) for each ITT was shown on the right (n = 6 mice in A/A, n = 8 in A/G and G/G). **e-f** Representative immunofluorescence staining of E18.5 pancreas with INS and GCG (**e**) and statistical analysis (**f**) of INS+/GCG+ polyhormonal cells percentage in each field under the microscope (n = 17 in A/A and n = 32 in G/G). The arrows indicate the polyhormonal cells. Scale bar, 20 μm. **g** Fasting blood glucose levels of each group after STZ injection (n = 4 mice in STZ A/A, STZ G/G and solvent G/G, n = 6 in STZ A/G and solvent A/G, n = 3 in solvent A/A, n = 6 in solvent A/G). **h** Quantification of the random blood glucose of each genotype after HFD (n = 3 mice). **i-j** Representative immunofluorescence staining of adult mice islets with Insulin (INS) and Glucagon (GCG) (**i**) and statistical analysis (**j**) of β cells and α cells percentage in HFD mice of each genotype (n = 22 in A/A, n = 19 in G/G). Scale bar, 20 μm. Data are presented as the mean ± SD. Statistical significance was determined using the unpaired, two-tailed t-test in **d**, **f**, **g**, **j** (ns not significant).

Supplementary Figure 8

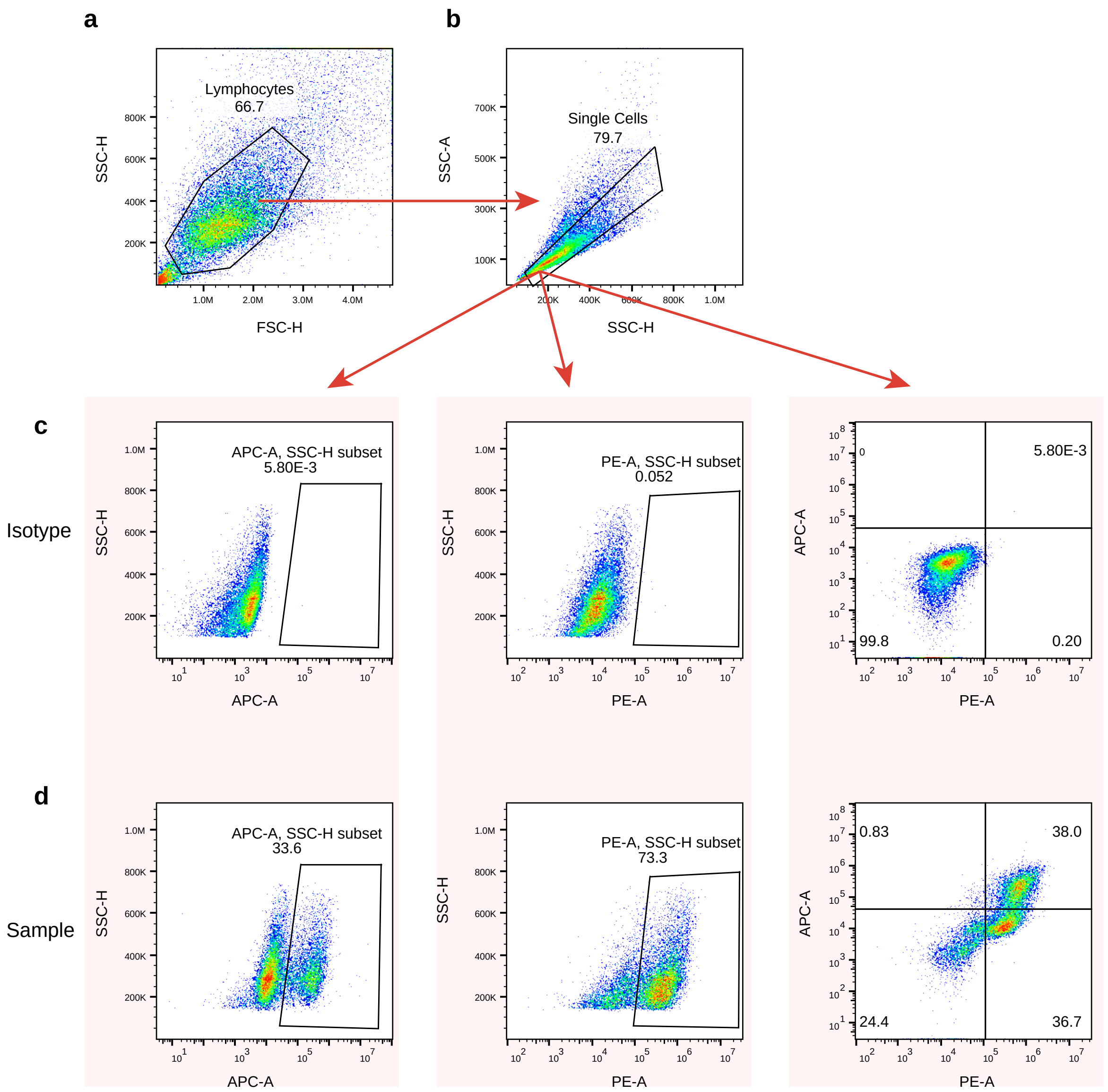

Supplementary Figure 8: Representative flow cytometry plots and gating strategy. **a** A cell population was selected based on size (FSC-H) and granularity (SSC-H) to exclude cell debris. **b** Doublets and multiplets were excluded from the population using side scatter area (SSC-A) and side scatter height (SSC-H), resulting in a population of single cells. **c-d** The single cells were then analyzed using APC-A and PE-A, with populations positive for either marker or both visualized on a two-parameter density plot of PE-A versus APC-A. Based on this plot, a gate was set for the isotype control, showing cells negative for PE-A and APC-A (**c**).

Supplementary Table 1: Primers for qRT-PCR analyses

Primer name	Forward primer	Reverse primer
GAPDH	AATGAAGGGGTCATTGATGG	AAGGTGAAGGTCGGAGTCAA
OCT4	CAAAGCAGAAACCCTCGTGC	TCTCACTCGGTTCTCGATACTG
SOX2	GTCATTTGCTGTGGGTGATG	AGAAAAACGAGGGAAATGGG
NANOG	CCCCAGCCTTTACTCTTCCTA	CCAGGTTGAATTGTTCCAGGTC
SOX17	GCATGACTCCGGTGTGAATCT	TCACACGTCAGGATAGTTGCAGT
FOXA2	GGAGCAGCTACTATGCAGAGC	CGTGTTCATGCCGTTCATCC
GATA4	CAGGCGTTGCACAGATAGTG	CCCGACACCCCAATCTC
GATA6	AGTTCCTACGCTTCGCATCCCTTC	TGAACAGCAGCAAGTCCTCCCA
PDX1	TTAGGATGTGGACGTAATTCCTGTT	GGCCACTGTGCTTGTCTTCA
INS	GCAGCCTTTGTGAACCAACAC	CCCCGCACACTAGGTAGAGA
GCG	CTGAAGGGACCTTTACCAGTGA	CCTGGCGGCAAGATTATCAAG
SST	CCCAGACTCCGTCAGTTTCT	ATCATTCTCCGTCTGGTTGG
NKX6-1	AGACCCACTTTTTCCGGACA	CCAACGAATAGGCCAAACGA
PTF1A	CAGGACACTCTCTCTCATGGA	TGGTGGTTCGTTTTCTATGTTGT
RXRA	ACAAGACGGAGCTGGGCTG	GGCTGCTCTGGGTACTTGTGC
UCP1	CTTCAGCGGCAAATCAGCTC	TCTTGCTTCCTAAACTAGGTGCT
SREBF1	CCTTGCATTTTCTGACACGCT	CAAGCTGTACAGGCTCTCCC
SCD	TGCGATATGCTGTGGTGCTT	TTGTGGAAGCCCTCACCCAC

Supplementary Table 2: Primers for ChIP-qPCR analyses

Primer name	Forward primer	Reverse primer
Allele A	CATGGTTGCTTAAACCGGCG	CATTGTGGGGTGCAGAGACT
Allele G	CATGGTTGCTTAAACCGGCG	CATTGTGGGGTGCAGAGACC
SREBF1	GGTTGGGGTTACTAGCGGAC	GTGTTGGGCCAGGACTTCTC
FOXA2	TGCCCACAGCATTTCGTAAC	GCCCCATCATTGATTCCTGG
peak1	CCTGTCCTCTGGCCAAACTG	AGGGGTAGGGAGTGATGCAA
peak2	GTCTCTGGCCGCATCTTGTG	AACTGGTGCTTCCCATGAAGA

Supplementary Table 3: Sequence of EMSA probes.

Probe name	Forward	Reverse
Allele-A	GGAAAAAAAGGAGGGGGGA GAGTCTCTGCACCCCACAAT-biotin	ATTGTGGGGTGCAGAGACTCTC CCCCCTCCTTTTTTTCC-biotin
Allele-G	GGAAAAAAAGGAGGGGGGA GGGTCTCTGCACCCCACAAT-biotin	ATTGTGGGGTGCAGAGACCCTC CCCCCTCCTTTTTTTCC-biotin
