# Supplementary material for: A noncoding variant confers pancreatic differentiation defect and contributes to diabetes susceptibility by recruiting RXRA
Source: Nat Commun. 2024 Nov 12;15:9771. doi: 10.1038/s41467-024-54151-y (PMC11557932; doi:10.1038/s41467-024-54151-y)
Supplement: Supplementary file 3 — Reporting Summary [file 41467_2024_54151_MOESM3_ESM.pdf]

Corresponding author(s): Wei Jiang
Last updated by author(s): Oct 21, 2024

Reporting Summary

Nature Portfolio wishes to improve the reproducibility of the work that we publish. This form provides structure for consistency and transparency in reporting. For further information on Nature Portfolio policies, see our Editorial Policies and the Editorial Policy Checklist.

Statistics

For all statistical analyses, confirm that the following items are present in the figure legend, table legend, main text, or Methods section.

n/a	Confirmed	
☐	☒	The exact sample size (*n*) for each experimental group/condition, given as a discrete number and unit of measurement
☐	☒	A statement on whether measurements were taken from distinct samples or whether the same sample was measured repeatedly
☐	☒	The statistical test(s) used AND whether they are one- or two-sided<br>*Only common tests should be described solely by name; describe more complex techniques in the Methods section.*
☐	☒	A description of all covariates tested
☐	☒	A description of any assumptions or corrections, such as tests of normality and adjustment for multiple comparisons
☐	☒	A full description of the statistical parameters including central tendency (e.g. means) or other basic estimates (e.g. regression coefficient) AND variation (e.g. standard deviation) or associated estimates of uncertainty (e.g. confidence intervals)
☐	☒	For null hypothesis testing, the test statistic (e.g. *F*, *t*, *r*) with confidence intervals, effect sizes, degrees of freedom and *P* value noted<br>*Give P values as exact values whenever suitable.*
☒	☐	For Bayesian analysis, information on the choice of priors and Markov chain Monte Carlo settings
☒	☐	For hierarchical and complex designs, identification of the appropriate level for tests and full reporting of outcomes
☒	☐	Estimates of effect sizes (e.g. Cohen's *d*, Pearson's *r*), indicating how they were calculated

Our web collection on statistics for biologists contains articles on many of the points above.

Software and code

Policy information about availability of computer code

Data collection	Immunostaining images were captured using Olympus IX53.
Data analysis	GraphPad Prism 9 was used for graphs and statistics. ImageJ was used for quantification of signals in Immunostaining. FlowJo (v10.8.1) was used to analyze FACS data. Adobe Illustrator 2021 was used to prepare figures.<br>Hisat2 (2.1.0) was used for alignment RNA-seq data. Featurecounts (1.6.3) was used for raw counts.DEseq2 was used for finding differential genes. g:Profiler (https://biit.cs.ut.ee/gprofiler/gost) was used for GO analysis. bowtie2 (2.3.4.3) was used for alignment CHIP-seq data. MASC2 (2.2.6) was used for peak calling. clusterProfiler (4.0.2) was used for peak annotation. Deeptools (3.3.0) was used for peak signal visualization.

For manuscripts utilizing custom algorithms or software that are central to the research but not yet described in published literature, software must be made available to editors and reviewers. We strongly encourage code deposition in a community repository (e.g. GitHub). See the Nature Portfolio guidelines for submitting code & software for further information.

Data

Policy information about availability of data

All manuscripts must include a data availability statement. This statement should provide the following information, where applicable:

- Accession codes, unique identifiers, or web links for publicly available datasets
- A description of any restrictions on data availability
- For clinical datasets or third party data, please ensure that the statement adheres to our policy

All the RNA-seq have been deposited in the Gene Expression Omnibus and the accession number for the reported in this work is GSE249854.

Human research participants

Policy information about studies involving human research participants and Sex and Gender in Research.

Reporting on sex and gender	*Use the terms sex (biological attribute) and gender (shaped by social and cultural circumstances) carefully in order to avoid confusing both terms. Indicate if findings apply to only one sex or gender; describe whether sex and gender were considered in study design whether sex and/or gender was determined based on self-reporting or assigned and methods used. Provide in the source data disaggregated sex and gender data where this information has been collected, and consent has been obtained for sharing of individual-level data; provide overall numbers in this Reporting Summary. Please state if this information has not been collected. Report sex- and gender-based analyses where performed, justify reasons for lack of sex- and gender-based analysis.*
Population characteristics	*Describe the covariate-relevant population characteristics of the human research participants (e.g. age, genotypic information, past and current diagnosis and treatment categories). If you filled out the behavioural & social sciences study design questions and have nothing to add here, write "See above."*
Recruitment	*Describe how participants were recruited. Outline any potential self-selection bias or other biases that may be present and how these are likely to impact results.*
Ethics oversight	*Identify the organization(s) that approved the study protocol.*

Note that full information on the approval of the study protocol must also be provided in the manuscript.

Field-specific reporting

Please select the one below that is the best fit for your research. If you are not sure, read the appropriate sections before making your selection.

☒ Life sciences ☐ Behavioural & social sciences ☐ Ecological, evolutionary & environmental sciences

For a reference copy of the document with all sections, see nature.com/documents/nr-reporting-summary-flat.pdf

Life sciences study design

All studies must disclose on these points even when the disclosure is negative.

Sample size	The number of samples (at least three independent biological replicates except for RNA-seq, which was done in two independent replicates) used in each experiment is based on previous experience.
Data exclusions	No data were excluded for the analysis.
Replication	The results were reproduced at least three times as independent biological replicates unless mentioned specifically.
Randomization	n/a
Blinding	The study involved unbiased quantification and analysis for immunostaining, and gene expression data sets. There was no expected outcome prior to the analysis, and blinding is not relevant.

Reporting for specific materials, systems and methods

We require information from authors about some types of materials, experimental systems and methods used in many studies. Here, indicate whether each material, system or method listed is relevant to your study. If you are not sure if a list item applies to your research, read the appropriate section before selecting a response.

March 2021

Materials & experimental systems

n/a	Involved in the study
☐	☒ Antibodies
☐	☒ Eukaryotic cell lines
☒	☐ Palaeontology and archaeology
☐	☒ Animals and other organisms
☒	☐ Clinical data
☒	☐ Dual use research of concern

Methods

n/a	Involved in the study
☒	☐ ChIP-seq
☐	☒ Flow cytometry
☒	☐ MRI-based neuroimaging

Antibodies

Antibodies used

anti-SOX17 (1:200, R&D, Cat#AF1924), anti-OCT4 (1:200, SANTA CRUZ, Cat#sc-5279), anti-PDX1 (1:200, R&D, Cat#AF2419), anti-NKX6-1 (1:300, ABclonal, Cat#A20419), anti-NKX6-1 (1:400, Cell Signaling Technology, Cat#54551), anti-FOXA2 (1:200, R&D, Cat#AF2400), anti-RXRA (1:300, Cell Signaling Technology, Cat#3085), anti-C-peptide (1:400, DSHB, Cat#GN-ID4), anti-Glucagon (1:500, Servicebio, Cat#GB11097) and anti-Glucagon (1:500, Servicebio, Cat#GB11335), anti-NKX6-1 (1:500, DSHB, Cat# F64A6B4), anti-PDX1 (1:500, R&D, Cat#AF2419) and anti-FOXA2 (1:1500, Cell Signaling Technology, Cat#8186) anti-SST (ZAGC-BIO, Cat#EP130/ZA-0567) were used in immunofluorescence assay.
anti-SOX17 (1:500, R&D, Cat#AF1924), anti-PDX1 (1:500, R&D, Cat#AF2419), anti-NKX6-1 (1:500, DSHB, Cat# F64A6B4), anti-C-peptide (1:800, DSHB, Cat#GN-ID4), anti-Glucagon (1:800, Servicebio, Cat#GB11097) and anti-Glucagon (1:500, Servicebio, Cat#GB11335) were used in flow cytometry analysis.
anti-RXRA (Cell Signaling Technology, Cat#3085) and anti-FOXA2 (R&D, Cat#AF2400) were used in ChIP-qPCR.

Validation

All the primary antibodies are commercial antibodies, validated by the manufacturers.

Eukaryotic cell lines

Policy information about cell lines and Sex and Gender in Research

Cell line source(s)	Human iPSC line PGP1 is described in Wang, G. et al (2014); HUES8 is banked as NIHhESC-09-0021.
Authentication	Routine quality control by microscopy morphology, immunostaining for pluripotent markers.
Mycoplasma contamination	All cell lines were confirmed as mycoplasma-negative before experiments.
Commonly misidentified lines (See ICLAC register)	No commonly misidentified line was used.

Animals and other research organisms

Policy information about studies involving animals; ARRIVE guidelines recommended for reporting animal research, and Sex and Gender in Research

Laboratory animals	C57BL/6J mouse (Ms musculus) was used. Knock-in C57BL/6J mice carrying the rs6048205 allele-G were constructed commercially (Shanghai Model Organisms Center Inc.). Male mice of each genotypes ranging from 2 weeks to 10 weeks were used for different purposes.
Wild animals	The study did not involve wild animals.
Reporting on sex	Since the glucose homeostasis of the two mice were similar, to eliminate the interference of estrous cycle and hormonal fluctuations in female mice, only male mice were used in subsequent experiments.
Field-collected samples	The study did not involve samples collected from the field.
Ethics oversight	All animal experiments were performed in accordance with the guidelines of Medical Research Institute animal care and use committee, Wuhan University.

Note that full information on the approval of the study protocol must also be provided in the manuscript.

Flow Cytometry

Plots

Confirm that:

☒ The axis labels state the marker and fluorochrome used (e.g. CD4-FITC).

☒ The axis scales are clearly visible. Include numbers along axes only for bottom left plot of group (a 'group' is an analysis of identical markers).

☒ All plots are contour plots with outliers or pseudocolor plots.

☒ A numerical value for number of cells or percentage (with statistics) is provided.

Methodology

Sample preparation	Cells were digested into single cells by 0.05% trypsin-EDTA and suspended in PBS contain 2% FBS. Cells were collected by centrifuging at 1000 rpm for 5 minutes at 4 °C, and then re-suspended and fixed according to the manufacturer's instructions of Transcription Factor Buffer Set (BD, Cat#562574). Fixed cells were washed and incubated with diluted primary antibody overnight at 4 °C. Then the cells were washed and incubated with diluted secondary fluorescent antibodies in dark for 2 hours at room temperature. Primary antibodies and secondary fluorescent antibodies were diluted in 1x Perm/Wash solution. After wash, cells were resuspended in PBS.
Instrument	flow cytometer (BD LSRFortessaX20 or ACEA NovoCyte)
Software	FlowJo (v10.8.1)
Cell population abundance	10000 cells after excluding cell debris.
Gating strategy	A lymphocyte gate was defined first from FSC-A vs SSC-A. Singlet gates were then defined on SSC-A vs SSC-H. Additional gating was performed as described in the figures for the individual experiments.

☒ Tick this box to confirm that a figure exemplifying the gating strategy is provided in the Supplementary Information.
